# Superficial Grain Refinement of 316L Stainless Steel by Rolling with Rough Rolls

**DOI:** 10.3390/ma16196416

**Published:** 2023-09-26

**Authors:** Yasmin Maril, Carlos Camurri, Oscar Zapata-Hernández, Claudia Carrasco, Marisol Maril

**Affiliations:** 1 Department of Materials Engineering, University of Concepción, Edmundo Larenas 315, Concepción 4070415, Chile; ccarrascoc@udec.cl (C.C.); marisolmaril@udec.cl (M.M.); 2 FIME-Centro de Investigación e Innovacion en Ingenieria Aeronautica (CIIIA), Universidad Autonoma de Nuevo Leon, Av. Universidad S/N, Ciudad Universitaria, San Nicolás de los Garza 66455, Mexico

**Keywords:** cold rolling, rough rolls, surface nanograins, recrystallization

## Abstract

This study presents a novel approach to producing superficial micro- and nanostructures using a cold rolling process with rough rolls, followed by low-temperature annealing. The proposed technique attempts to recreate the superficial deformation occurring in the sandblasting process. It allows for the generation of an inhomogeneous network, or tangle, of high-deformation zones on the material’s surface that act as nucleation centers during the subsequent annealing process. However, the proposed method has a significant advantage over sandblasting: it is a continuous process with high productivity. An austenitic stainless-steel sheet, previously normalized, was used as the raw material. The samples were cold rolled using rough rolls (rhombic-based pyramids of 2.08 mm, 1.04 mm, and 1.5 mm in length, width, and height, respectively) and annealed at temperatures between 200 °C and 400 °C for one hour. An optical and electronic microstructure analysis showed the presence of small, heterogeneously distributed surface grains of 200–300 nm in diameter. Finite element analysis revealed significant deformation that was inhomogeneous and likely responsible for the uneven distribution of the recrystallized grains. Additionally, surface nanohardness results showed a 20% increase with respect to the central zone of the material. Finally, wear tests of the treated samples showed lower wear than samples rolled with conventional rolls.

## 1. Introduction

The ever-increasing demand for advanced mechanical properties in metals and alloys has led several researchers to focus on this task. It is well known that strength and wear resistance are improved by grain size reduction [1,2]. In addition to this, it has been found that grain refinement can increase a large number of additional properties of different metals and alloys, such as corrosion resistance [3,4], creep resistance [5,6], pitting resistance [7], and resistance to cavitation and cavitation erosion [8,9], among others. 

Strengthening derived from grain refinement is usually governed by the Hall–Petch equation, which indicates that smaller grain sizes produce higher yield stress. However, in many cases, damage or maximum stresses occur near the surface regions, causing many of the failures of a workpiece, such as wear, fatigue, and corrosion, to initiate on the surface. Because of this, the microstructure and properties of the surfaces of structural materials can play an essential role in failure control. Many studies have focused on improving the surface properties of materials by refining surface grains. Over the last few decades, several surface nanostructuring techniques have been successfully developed. They have been successful in forming ultrafine grains (UFGs) and nanograins (NGs) on the surfaces of different classes of metallic materials, including steel, aluminum, copper, nickel, titanium, and other alloys [10,11,12]. 

Along with developing these techniques, studies have demonstrated that nanocrystalline surfaces bring many enhancements, such as prolonged fatigue life, to ordinary metallic materials because the surface layer impedes the movement of dislocations, delaying crack initiation [13]. Nevertheless, the contributions of these techniques go far beyond that and can enhance hardness, corrosion [14], and other tribological properties. Among the most reproducible processes that refine superficial grains are severe shot peening (SSP) [15,16,17], ultrasonic shot peening [18,19], high-energy shot peening [20,21], surface mechanical attrition (SMA) [22,23], and ultrasonic impact peening [24,25]. 

The common feature of all of the aforementioned surface nanostructuring methods is that the top surface layer is subjected to severe plastic deformation at high strain rates, creating an NG layer on and just below the surface and exposing deeper material to compressive residual stress. Other studies have focused on the same goal by developing methods involving the deformation–recrystallization sequence. Most of these studies focus on sandblasting [26,27]. However, there are other processes that make use of this sequence [28]. In this technique, the specimens are sandblasted and subsequently annealed at low temperatures. The sandblasted surface layer is heavily plastically deformed and consequently has high-density dislocations. After the annealing process, a structure with NGs is obtained. For example, Wang et al. [29] demonstrated the generation of a nanocrystalline surface layer on AISI 304 stainless steel by sandblasting with 0.21–0.30 mm silica grains followed by annealing at 350 °C for one hour. This process produced 20 nm grains, and the presence of this surface layer considerably improved the steel’s corrosion resistance and mechanical properties. 

Despite successfully forming surface NGs, none of the abovementioned techniques are compatible with ongoing processes or for processing the most extensive surfaces due to their low operating speeds, where the major limitation for the latter is the requirement to upscale the size of the parts, including the length and cross-sectional area. To overcome these drawbacks, and considering the industrial limitations of classical nanosurface processes, some years ago, a rolling process with four to eight passes using rough rolls followed by annealing at low temperature was proposed to generate superficial micro- and even nanostructures to treat the largest surfaces in a high-productivity manner [30]. This process simulates the superficial deformation occurring in sandblasting and could generate a dense, complex, high field and tangles of superficial deformations acting as “infinite” nucleation centers during the subsequent low-temperature annealing, ultimately giving rise to surface micrograins or NGs. Preliminary results [30] indicated that it was a feasible technique, as it enables the production of surface grains smaller than those generated through traditional rolling methods. Therefore, this study aimed to enhance our understanding of this proposed grain refinement technique through both experiments and simulations. In particular, it is intended to study the effect of different roller roughnesses on the generation of these surface grains and variables of the rolling process, as well as the correlation between the deformation field and the grains obtained on the surface. Additionally, the surface mechanical properties of samples rolled with rough rolls, mechanisms, and wear rates are analyzed, comparing them with those of a conventional material.

## 2. Materials and Methods

The following section furnishes details regarding the materials employed and the methods applied in this research. It outlines the sample preparation process and the subsequent characterization, encompassing property assessment and mathematical analysis.

### 2.1. Materials

Since stainless steel was used in many previous works, AISI 316 sheets (Acerinox, Cádiz, Spain) with a thickness of 5 mm were used in this work as the raw material. The nominal composition of the stainless-steel material is given by the ASTM A240 [31] and ASME SA-240 [32] standards and is listed in Table 1. The sheets were cut into plates measuring 5 cm × 6 cm. In order to get a homogeneous structure, the plates were previously normalized at 900 °C for two hours.

### 2.2. Rolling and Annealing Treatment

The plates were cold rolled in six passes with an absolute reduction of 0.5 mm/pass using a 7.5 kW Joliot two reversible rolling mill equipped (Joliot, France) with rolls that were 200 mm wide and 127 mm in diameter. To obtain a rough surface on the rolls, rhombic-based pyramids were machined on the surface, the dimensions of which were 2.08 mm long, 1.04 mm wide, and 1.5 mm high. After the rolling process, the samples were annealed at different temperatures, 200 °C, 300 °C, and 400 °C, for one hour to allow the recrystallization process. A batch-type muffle furnace was used to carry out this treatment. In this particular case, substantial pre-deformation of about 60% occurs. This cold deformation introduces dislocations into the crystalline structure of the material, leading to an increase in internal energy which, in turn, favors the formation of new grains at lower energy levels, enabling the recrystallization process to occur at these reduced temperatures.

### 2.3. Morphological and Structural Characterization

Cross sections of the rolling samples for morphological and structural observations were mechanically ground with SiC paper down to an average scratch size of 8 µm, finely polished with diamond paste and aluminum oxide (0.05 µm), then rinsed ultrasonically in ethanol. Each sample was then etched in a solution containing a mixture of nitric acid and hydrochloric acid in a molar ratio of 1:3 (aqua regia) to characterize the as-received material’s microstructure. The analysis was carried out by optical microscopy using Olympus GX51 equipment (Tokyo, Japan). A confocal laser scanning microscope Olympus LEXT (Tokyo, Japan) was used to observe and compare the treated surface morphology using rough rolls versus conventional rolls.

In order to study the grain sizes of the near-surface layer, the samples were electro-etched using 60% nitric acid and 40% water with a voltage of 8–9 A/cm^2^ for 120 s; this technique delineates grain boundaries without revealing annealing twins and thereby makes grain size determination much simpler. For this purpose, scanning electron images were gathered on the VEGA3 TESCAN microscope Brno, Czech Republic at 20 kV. In addition, electron backscatter diffraction (EBSD) analysis was conducted with a field emission Gun Hitachi SU-70 (Tokyo, Japan) scanning electron microscope (SEM) with an EDAX system (Berwyn, Pennsylvania, United States) and the OIM-TSL 7.0 analysis software.

### 2.4. FEM Model

**Figure 1:**
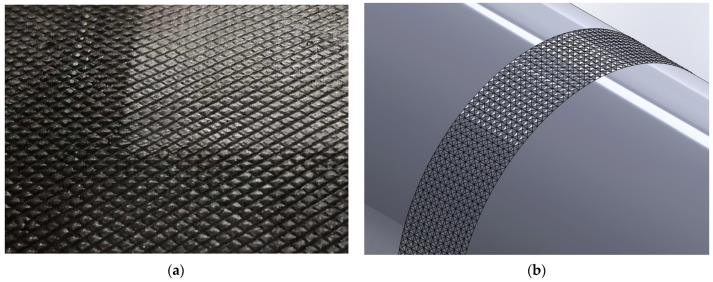
Roll surface: (**a**) experimental and (**b**) simulated with Simufact.

To analyze the deformation field on the material’s surface and the mechanical variables associated with the process, a 3D finite element model (FEM) using Simufact software package version 19.0 was employed. The rolling setup consisted of two rolls and a plate as the workpiece. The rolls had the exact dimensions used in the experimental part and the dimensions of the workpiece were 60 mm long, 25 mm wide, and 5 mm high. Moreover, the analysis considered sticking friction at the contact roll plate according to Von Mises model. The simulated roughness on the roll’s surface was created by utilizing square-based pyramids of 2.5 mm in length and 0.25 mm in height. At this stage, it was chosen to simulate pyramids of lower height than experimental pyramids to produce a lower roughness in the modeling approach, as the computational demands of simulating high pyramids can be significant. Working with more manageable dimensions ensures that the software can efficiently produce results. Figure 1a shows the roll surface utilized in the experimental analysis, while Figure 1b illustrates the one simulated using Simufact.

**Figure 2:**
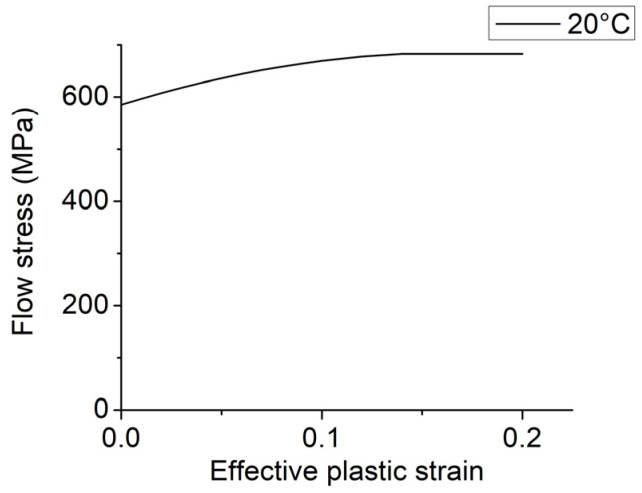
Flow curve of the 316L stainless steel at 20 °C.

**Figure 3:**
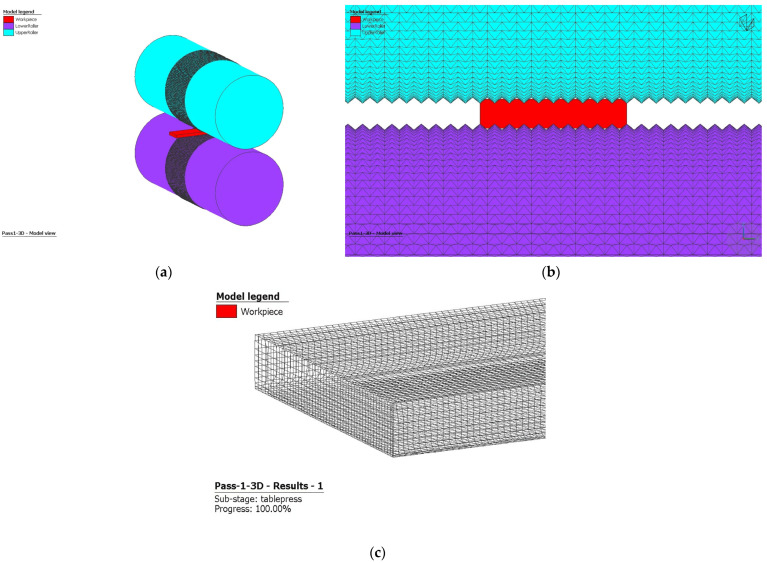
(**a**) FEM, (**b**) geometrical configuration, and (**c**) workpiece mesh.

To characterize the mechanical behavior of the 316 stainless steel, flow curves were obtained from MatILDa^®^ software, which is embedded in Simufact software. These curves provide a reliable foundation for material calculations based on true strain, strain rate, and forming temperature, presented either in tabular or analytical form. Figure 2 showcases the relationship between flow stress and effective plastic strain at a temperature of 20 °C for the 316L steel grade studied. Figure 3 shows the finite element model of the first pass of rolling with high roughness on the surface of the rolls.

### 2.5. Wear Resistance and Nanoindentation Characterization

A wear test was performed under unlubricated conditions using a TRM 1000 tribometer. The standard load was 30 N, the velocity was 0.1 m/s, and the distance was 200 m. Secondary electron imaging SEM was used to determine the mechanism that had produced the wear observed on the surface exposed during the wear tests. To measure hardness as a function of the depth to the top surface, nanoindentation was performed using a Hysitron TI Premier nanotriboindenter. A diamond Berkovich tip was used, and the loading–holding–unloading technique was adopted to measure the hardness at different depths. For each indent, three loading–holding–unloading segments (25 s, 5 s, and 2 s, respectively) were performed using load control mode and an applied load of 5000 µN.

## 3. Results

### 3.1. Microstructural Analysis

**Figure 4:**
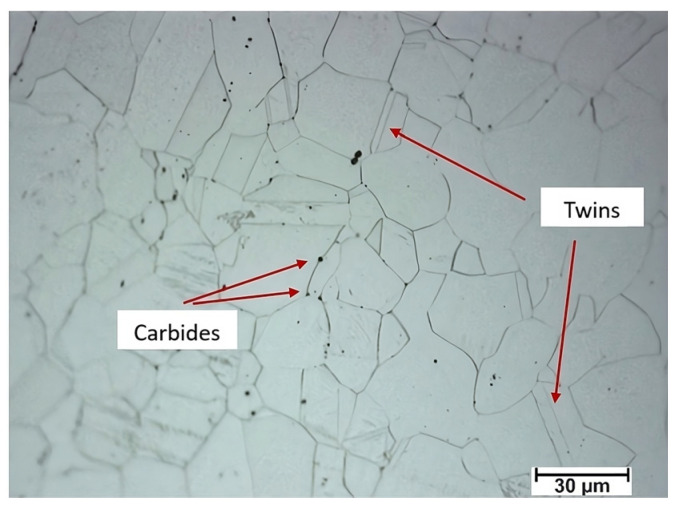
The original microstructure of the 316 stainless steel before rolling.

Figure 4 shows the microstructure characteristics of the austenitic stainless steel 316L, which was previously annealed at 900 °C for two hours before rolling. Considering this alloy’s chemical composition, the microstructure consists of austenite grains (30 µm), which may exhibit twinning, as in this case. We can also observe the formation of complex carbides.

**Figure 5:**
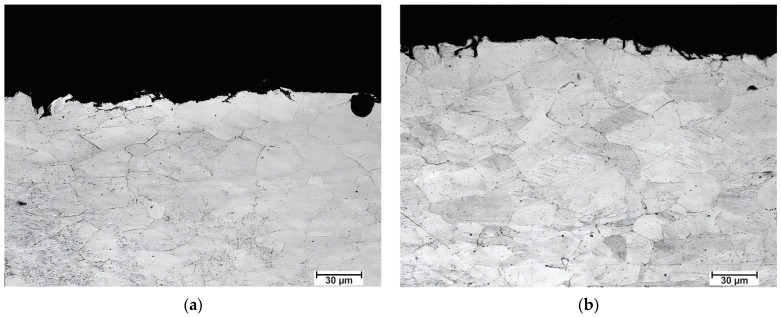
Micrographs of a sample rolled with conventional rolls and annealed at (**a**) 300 °C and (**b**) 400 °C for one hour.

Figure 5 shows micrographs of the samples rolled using conventional rolls after annealing. In Figure 5a, the sample was annealed at 300 °C, while in Figure 5b, the sample was annealed at 400 °C, both for one hour. Recrystallized grains can be observed without deformed grains. No surface refinement of the grain sizes is observed; instead, it is possible to appreciate a homogeneous grain size distribution, with both samples having similar grain sizes of 30–35 µm.

**Figure 6:**
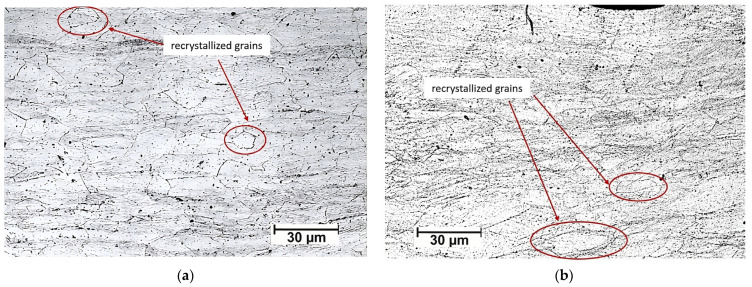
Micrographs of a sample rolled with rough rolls and annealed at 300 °C for one hour: (**a**) bulk and (**b**) near surface.

**Figure 7:**
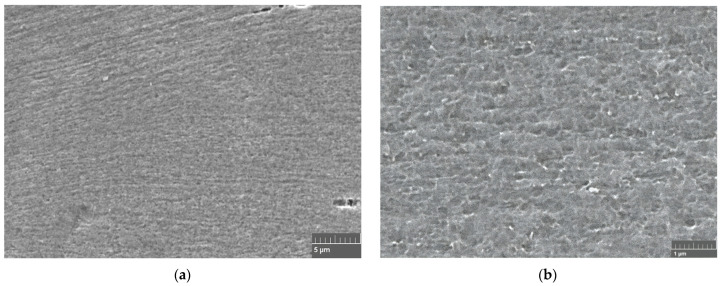
SEM images of a sample rolled with rough rolls and annealed at 400 °C for one hour. (**a**) 10,000× and (**b**) 25,000×.

Figure 6 shows a sample rolled using rough rolls and recrystallized at 300 °C for one hour. Note that the interior zone of this sample (Figure 6a) has grains of a similar size to all those observed in the samples rolled with conventional rolls. The presence of some recrystallized grains in the bulk has been highlighted in red circles. While, in Figure 6b, it is possible to observe both the surface and the sample’s bulk, it is possible to discern recrystallized grains distinctly marked with red circles within the bulk. Conversely, when examining the surface, there is only a microstructural variation compared to the central region. It is important to note that discerning the presence of ultrafine or nanometric grains at this observation scale is not feasible. For this reason, Figure 7 shows the surface layer of a sample rolled using rough rolls and recrystallized at 400 °C for one hour using scanning electron microscopy. In these images, it is possible to observe the presence of recrystallized grains oriented in the direction of lamination measuring approximately 200 nm.

**Figure 8:**
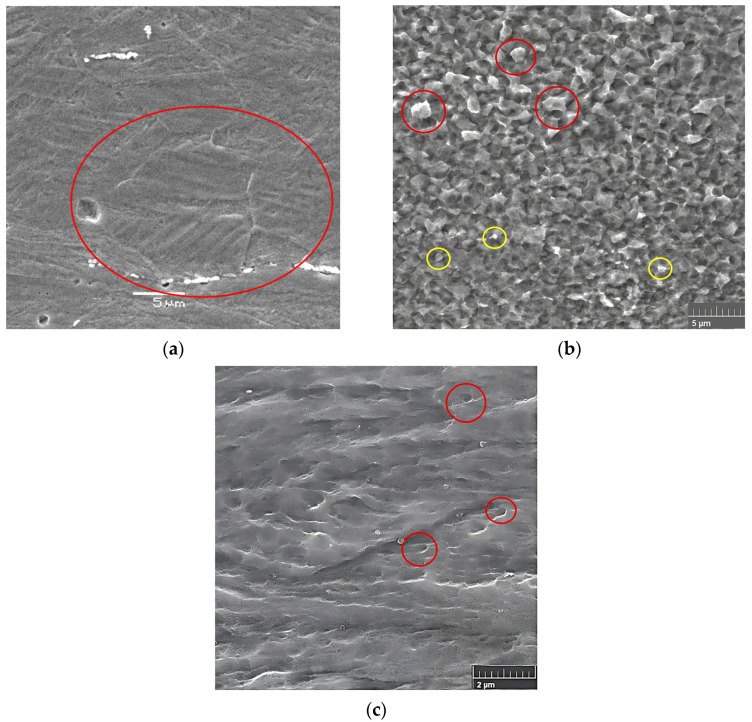
SEM images of a sample rolled with rough rolls and annealed at (**a**,**b**) 300 °C and (**c**) 400 °C, both for one hour.

Figure 8 presents SEM micrographs of the near-surface microstructure obtained for rolled samples with rough rolls followed by annealing at 300 °C and 400 °C for one hour. In Figure 8a, an evident feature is the presence of newly recrystallized grains measuring approximately 10 μm (enclosed in red circles). Figure 8b showcases even smaller grains, some of them measuring less than 1 μm (enclosed in yellow circles), with an average diameter of about 3 μm (enclosed in red circles), indicating heterogeneity in the distribution of the recrystallized grain sizes. A closer inspection of Figure 8c, specifically within the red circle, reveals the presence of recrystallized grains with an average size of approximately 200 nm. However, it can be also seen that the recrystallization of new grains occurs heterogeneously.

**Figure 9:**
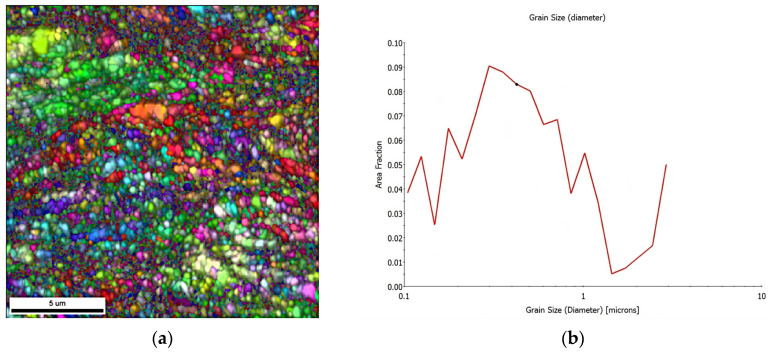
(**a**) EBSD images of a sample rolled with rough rolls and annealed at 300 °C for one hour. (**b**) Area fraction versus grain size.

Figure 9a shows images obtained by EBSD of near-surface areas. The partial generation of new recrystallized grains of nanometer size can be appreciated within them. As shown in previous figures, the generation of these grains appears partial and inhomogeneous. This phenomenon can be attributed to the fact that the deformation was not homogeneous on the surface, and therefore the recrystallization was not homogeneous either. The graph in Figure 9b further demonstrates this phenomenon, where the area fraction versus grain size is presented. It can be seen that a large fraction of the total area retains grains with sizes greater than 1 μm and those of nanometer sizes only occupy a small percentage of the total area.

### 3.2. FEM Model Analysis

The following sections and corresponding figures show and discuss the results obtained using Simufact in terms of plastic strain and rolling stresses and forces. 

#### 3.2.1. Plastic Strain

**Figure 10:**
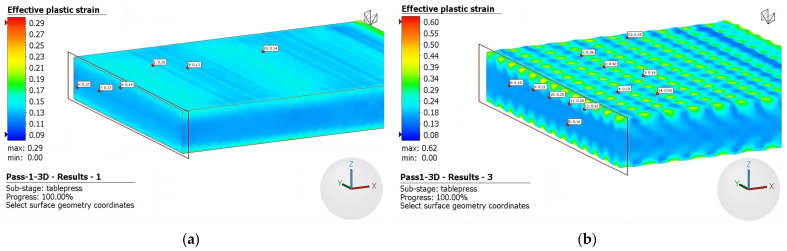
Effective plastic strain at first pass using (**a**) conventional and (**b**) rough rolls.

Figure 10 presents the results of the first rolling pass, which reduced the initial thickness from 5 mm to 4.5 mm, using a 3D deformation model. Figure 10a depicts the effective plastic strain obtained with conventional rolls, while Figure 10b shows the results obtained with rough rolls. As shown in Figure 10a, the maximum deformation corresponds to 0.29, while the minimum to 0.09. However, the minimum and maximum values identified in the front and rear areas of the plate do not provide a reliable representation of the overall behavior of the workpiece. The figure also presents an image magnification revealing a gradual variation from the center of the piece, exhibiting a deformation of 0.12, to the surface of the piece, where the strain reaches a value of 0.13–0.15, indicating the low inhomogeneity of the deformation. In Figure 10b, where rough rolls were used, the variation of deformations across the entire workpiece is much more pronounced. In the center of the piece, the deformation reaches values of 0.16, while on the surface, there are zones with similar strain values (indicated by blue areas) where the indentation does not significantly strain the piece, and other zones with high deformation, reaching values of up to 0.43 (indicated by red areas) due to the indentation effect of the rough rolls.

Recognizing that the minimum and maximum values fail to represent the overall behavior of the workpiece adequately, a comprehensive analysis was conducted using the same approach employed in the first-pass model. Detailed scrutiny of various regions of the workpiece, both at its center and on the surface, revealed deformation values that are documented in Table 2. An increase in the effective strain is observed as the number of passes increases, indicating a direct correlation between the number of passes and the extent of deformation experienced by the workpiece. This effect is particularly pronounced on the rough surface, due to the indentation effect.

**Figure 11:**
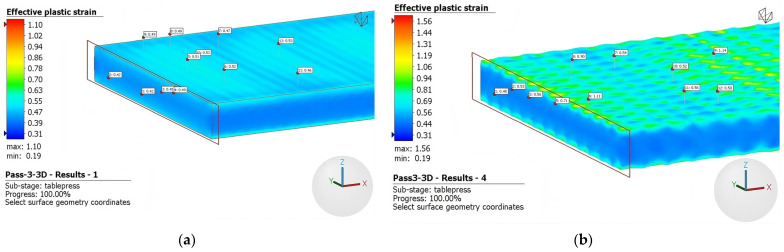
Effective plastic strain third pass using (**a**) conventional rolls and (**b**) rough rolls.

**Figure 12:**
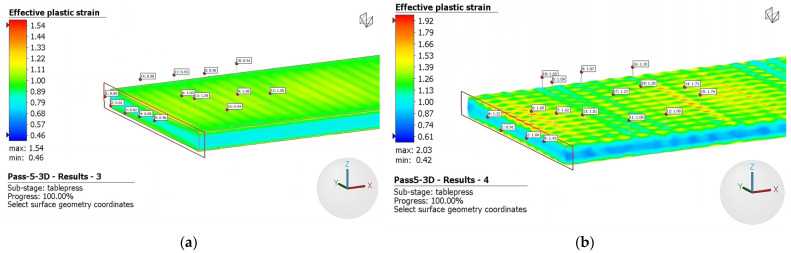
Effective plastic strain fifth pass using (**a**) conventional rolls and (**b**) rough rolls.

The results for the third and fifth rolling passes are depicted in Figure 11 and Figure 12, respectively. During the third pass, the plate’s thickness was reduced from 4 mm to 3.5 mm, while in the fifth pass the plate was reduced from 3.0 mm to 2.5 mm. As mentioned above, it can be seen graphically that deformation, which is primarily concentrated at the top of the surface, intensifies using rough rolls. Notably, there are distinct regions of inhomogeneity on the surface, characterized by significantly higher levels of deformation. This nonuniform deformation pattern arises from the discontinuous roughness imposed by the rough surface of the rolls. Consequently, a notable increase in the level of effective plastic strain is observed when comparing the fifth, third, and initial passes.

As seen above, Figure 8 and Figure 9 illustrate the limited occurrence of recrystallized surface grains, displaying a nonuniform and heterogeneous distribution. This phenomenology can be attributed to the indentation on the surface, which does not generate a uniformly distributed field of micro-deformations. Evidence of this effect is presented in Figure 10, Figure 11 and Figure 12, where regions adjacent to the indenter reveal a cluster of high surface deformations, potentially serving as nucleation centers.

#### 3.2.2. Stress

**Figure 13:**
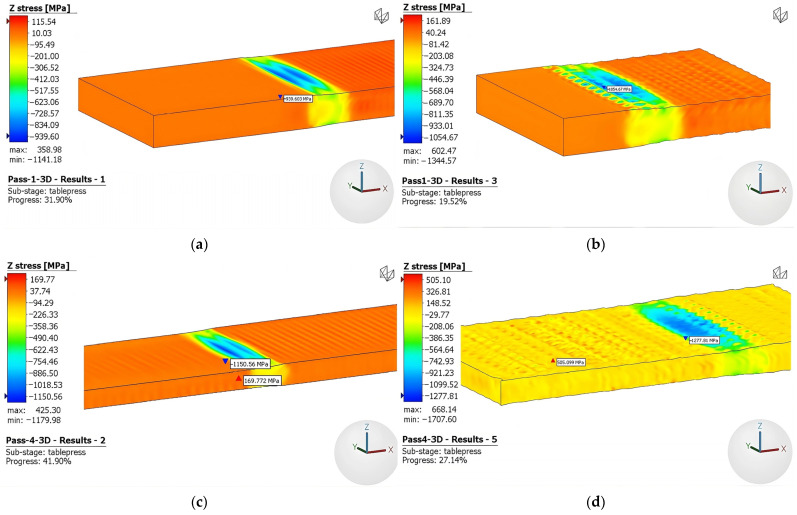

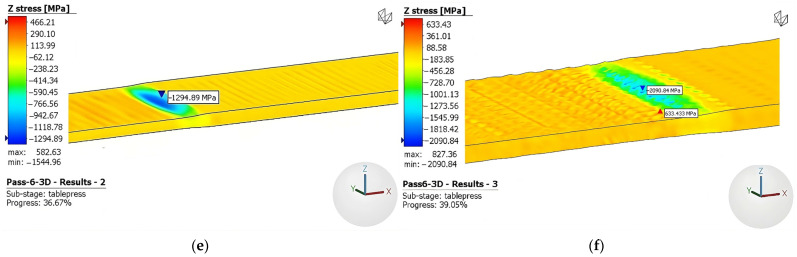
Effective stress 3D model: (**a**) first-pass conventional rolls, (**b**) first-pass rough rolls, (**c**) fourth-pass conventional rolls, (**d**) fourth-pass rough rolls, (**e**) sixth-pass conventional rolls, and (**f**) sixth-pass rough rolls.

To develop a comprehensive understanding of the pressure variation imparted to the sample during the rolling process, a thorough analysis was performed to examine the stress behavior along the vertical z-direction. The stress distribution in the z-direction of the models during the first pass, fourth pass, and final pass is illustrated in Figure 13. Note that negative values correspond to compressive stress, and as anticipated, the maximum compression occurs at the surface. The pressure increases with the number of passes due to strain hardening and the utilization of rough rolls amplifies this stress phenomenon.

#### 3.2.3. Rolling Forces

**Figure 14:**
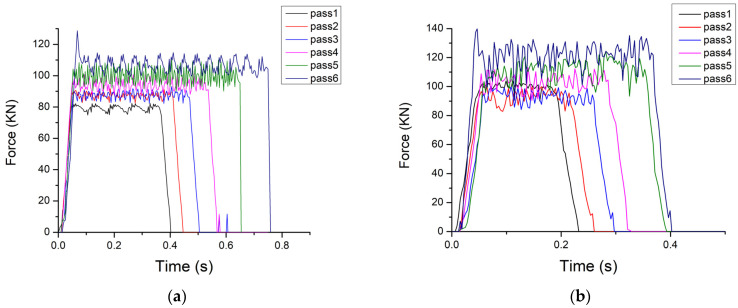
Force of all passes for the (**a**) conventional and (**b**) rough rolls.

Figure 14 depicts the rolling force for all simulated passes for conventional and rough rolls. The graph clearly illustrates a trend of progressively increasing force as the number of passes is increased. This phenomenon, together with the increase in the z-direction stress, can be attributed to the strain hardening effect that occurs with each subsequent pass, noting that the absolute reduction is constant in each pass and equal to 0.5 mm. Conversely, when using rough rolls, the rolling force values are higher compared to the conventional rolls. This increase is attributed to the indentation created by the rough surface of the roll.

### 3.3. Wear Resistance and Nanohardness Analysis

**Figure 15:**
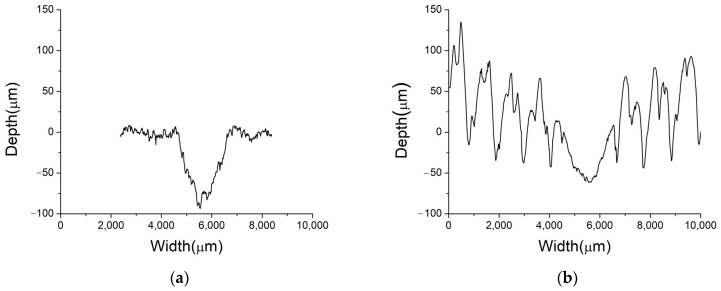
Wear groove profile of samples rolled with (**a**) conventional rolls, (**b**) rough rolls, and then annealed at 400 °C.

Figure 15 shows the profiles obtained from the wear grooves. Figure 15a shows the wear profile of a sample rolled using conventional rolls, and Figure 15b shows the results using rough rolls, both annealed at 400 °C. A comparison between the two rolling techniques reveals noticeable differences in the groove profiles, affecting both depth and width. Additionally, it is feasible to observe the roughness profile on the sides of the groove resulting from the wear test. Notably, when rough rolls are utilized, this leads to an increase in the overall surface roughness of the sample. Specifically, the depth and width of the groove caused by the sample rolled with conventional rolls measure approximately 88 µm and 2200 µm, respectively. On the other hand, when rough rolls are employed, these values decrease, with the depth measuring approximately 65 µm and the width around 2000 µm. These findings highlight the impact of the rolling technique on the resulting groove dimensions, with conventional rolls producing deeper and wider grooves compared to the rough rolls. Therefore, the use of rough rolls increases the wear resistance.

**Figure 16:**
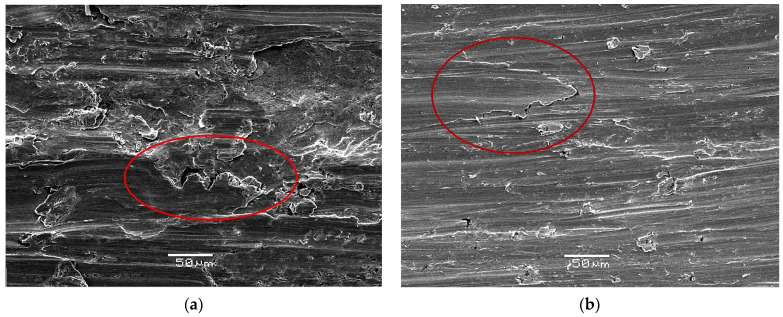
SEM images of the wear mechanism of samples rolled with (**a**) conventional rolls and (**b**) rough rolls.

Figure 16 shows images obtained by the SEM of the wear topography after the wear test. Figure 16a shows an image of the wear of a sample rolled using conventional rolls. The image shows an area (enclosed in red the circle) with lateral displacement of the metal due to plastic deformation corresponding to a mixture of wear mechanisms, including grooves caused by plowing and wedging. This behavior is typical of ductile materials. On the other hand, Figure 16b shows a sample that was rolled using rough rolls and then annealed at 400 °C. Characteristic cutting wear is seen and has been highlighted in the red circle, in which there is no material displacement towards the sides, as seen in the plowed groove, nor any accumulated material at the end of the groove, as occurs in wedge-type grooves, indicating that the surface has been completely removed. Therefore, the removal of the surface can be attributed to an increase in its mechanical properties on the surface as a result of rolling with rough rolls and the consequent smaller grain size induced in that zone.

**Figure 17:**
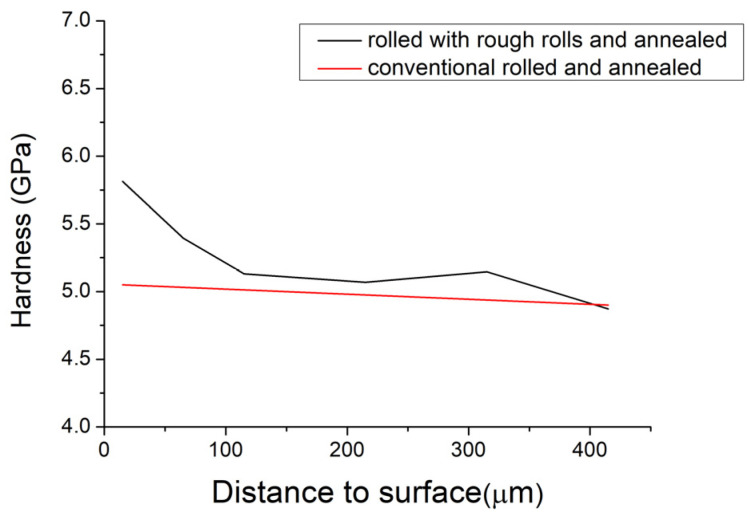
Curve of the nanohardness that corresponds to distance to the surface of rolled samples with conventional and rough rolls.

Figure 17 illustrates the nanohardness profile of a sample rolled with rough rolls and a sample rolled with conventional rolls, both annealed at 400 °C for 1 h, as a function of the distance from the surface. Notably, the nanohardness values exhibit the anticipated trend for the sample rolled with rough rolls, with higher values observed at the surface compared to the bulk of the material. Specifically, the nanohardness at the surface of the sample measures 5.8 GPa, while it decreases towards the bulk, reaching a nanohardness value of 4.9 GPa. On the other hand, the red curve represents the nanohardness values for a sample rolled with conventional rolls. It is possible to observe that the nanohardness values are very close on the surface and in bulk, reaching around 5 GPa. The findings in Figure 17 underscore the influence of surface treatment on the mechanical properties of the material. The decrease in nanohardness towards the bulk signifies an alteration in the microstructure resulting from grain refinement near the surface when rough rolls are used in the rolling process.

**Figure 18:**
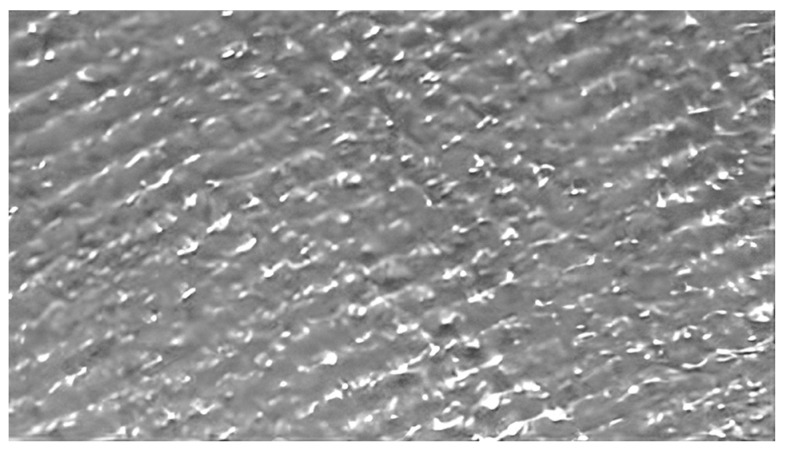
Specimen surface after 6 rolling passes using rough rolls.

Figure 18 displays the surface of a sample after undergoing six rolling passes. The image reveals the absence of micro-cracks and shows surface roughness resulting from using rough rolls during the rolling process. This phenomenon indicates the effectiveness of the process in achieving a micro-crack-free surface and highlights the desirable presence of controlled roughness at this stage of the process.

## 4. Conclusions

This study revealed that rolling with rough rolls yields micrometer- and even nanometer-sized surface grains through grain refinement by the process of deformation–recrystallization. 

Results from advanced microscopy techniques including SEM and EBSD reveal selective and localized recrystallization, leading to the formation of nanometer-sized grains in the near-surface region. This phenomenon occurs due to the induction of high surface deformation zones that act as nucleation centers for new grains during recrystallization. However, the high roughness of the rolls does not allow for a homogeneous field of high deformations to be obtained. As such, the surface NGs were present in an isolated and heterogeneous way.

The results of wear resistance and nanohardness tests allow one to conclude that the superficial grain refinement induced in the proposed process increases the material’s wear resistance, offering a promissory alternative for producing high-quality micro- and nanostructures with improved surface properties.

Mathematical simulation convincingly demonstrates that the selection between conventional and rough rolls has a noteworthy influence on deformation, stress levels, and rolling forces; showing that the above-mentioned mechanical properties increase when roughness increases.

## Figures and Tables

**Table 1:** Nominal chemical composition of the 316L stainless steel used in this study [33].

Element	C max	Cr	Ni	Mn Max	Si Max	P Max	S Max	Mo	Fe
Weight percent	0.03	16.00–18.00	10.00–14.00	2.00	1.00	0.045	0.03	2.00–3.00	Balance

**Table 2:** Effective plastic strain for the simulated models.

Pass 1	Conventional		Roughness	
	Bulk	Surface	Bulk	Surface
1	0.13	0.13–0.16	0.16	0.15–0.43
2	0.26	0.29–0.35	0.30	0.30–0.87
3	0.42	0.46–0.53	0.45	0.50–1.14
4	0.61	0.66–0.79	0.66	0.75–1.49
5	0.81	0.91–1.09	0.91	1.05–1.74
6	1.08	1.17–1.42	1.22	1.40–1.99

## Data Availability

All data are available in the manuscript or upon request to the corresponding author.
